# Supplementary figures and images for: Centrosome Movements Are TUBG1-Dependent
Source: Int J Mol Sci. 2023 Aug 24;24(17):13154. doi: 10.3390/ijms241713154 (PMC10488117; doi:10.3390/ijms241713154)

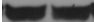

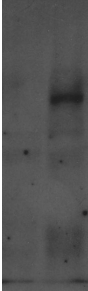

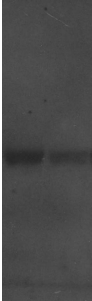

Supplement: Supplementary file 1 [file ijms-24-13154-s001.zip › supplementary_Files.pdf]
